# Mechano-stimulated modifications in the chloroplast antioxidant system and proteome changes are associated with cold response in wheat

**DOI:** 10.1186/s12870-015-0610-6

**Published:** 2015-09-11

**Authors:** Xiangnan Li, Chenglong Hao, Jianwen Zhong, Fulai Liu, Jian Cai, Xiao Wang, Qin Zhou, Tingbo Dai, Weixing Cao, Dong Jiang

**Affiliations:** National Engineering and Technology Center for Information Agriculture / Key Laboratory of Crop Physiology and Ecology in Southern China, Ministry of Agriculture, Nanjing Agricultural University, Nanjing, 210095 China; Faculty of Science, Department of Plant and Environmental Sciences, University of Copenhagen, Højbakkegaard Allé 13, DK-2630 Taastrup, Denmark

**Keywords:** Mechano-stimulation, Cold, Reactive oxygen species, Chloroplast, Wheat

## Abstract

**Background:**

Mechanical wounding can cause morphological and developmental changes in plants, which may affect the responses to abiotic stresses. However, the mechano-stimulation triggered regulation network remains elusive. Here, the mechano-stimulation was applied at two different times during the growth period of wheat before exposing the plants to cold stress (5.6 °C lower temperature than the ambient temperature, viz., 5.0 °C) at the jointing stage.

**Results:**

Results showed that mechano-stimulation at the Zadoks growth stage 26 activated the antioxidant system, and substantially, maintained the homeostasis of reactive oxygen species. In turn, the stimulation improved the electron transport and photosynthetic rate of wheat plants exposed to cold stress at the jointing stage. Proteomic and transcriptional analyses revealed that the oxidative stress defense, ATP synthesis, and photosynthesis-related proteins and genes were similarly modulated by mechano-stimulation and the cold stress.

**Conclusions:**

It was concluded that mechano-stimulated modifications of the chloroplast antioxidant system and proteome changes are related to cold tolerance in wheat. The findings might provide deeper insights into roles of reactive oxygen species in mechano-stimulated cold tolerance of photosynthetic apparatus, and be helpful to explore novel approaches to mitigate the impacts of low temperature occurring at critical developmental stages.

**Electronic supplementary material:**

The online version of this article (doi:10.1186/s12870-015-0610-6) contains supplementary material, which is available to authorized users.

## Background

Chilling temperature significantly affects the early growth of winter wheat plants causing considerable reduction of grain yield and is one of the major factors limiting growth and productivity of crops [1]. Cold induced photosynthesis inhibition results in a complex array of reactive oxygen species (ROS) generation, especially in chloroplasts [2]. Over-accumulation of ROS may cause rigidification and leakage of the cell membrane, and destabilization of protein complexes [1]. Recent proteomic studies have revealed differential expression of proteins in wheat exposed to cold stress [3, 4]. Among the down-regulated proteins due to cold stress, some key enzymes involved in Krebs cycle (isocitrate dehydrogenase, malate dehydrogenase) have been identified, together with many photosynthesis-related proteins (e.g. oxygen-evolving complex proteins, ATP synthase subunits, ferredoxin NADPH oxidoreductase, and some Calvin cycle enzymes) [3]. Proteomic analysis of spring freezing stress responsive proteins in leaves revealed an increased accumulation of stress defense proteins, including LEA-related COR protein, Cu/Zn superoxide dismutase, and ascorbate peroxidases, which may play crucial roles in enhancing tolerance to spring freeze stress in bread wheat [4]. In addition, proteomic analysis of wheat in response to prolonged cold stress showed reinforcement in expressions of enzymes involving in ascorbate recycling (dehydroascorbate reductase, ascorbate peroxidase) and involving in tetrapyrrole re-synthesis (glutamate semialdehyde aminomutase) [3].

Mechanical wounding can be caused by surrounding environmental factors, such as wind, rainstorms, and herbivores, and it has broad impacts on plants, including changes in morphogenetic characteristics, membrane potential [5], ROS, hormone signaling and gene expression [6]. Several alterations induced by mechanical wounding can allow plants to resist and acclimate to environmental stresses [6]. As previously observed in maize, bean, and rice, denser but smaller stomata in mechanically stimulated leaves could help plants to control transpirational water loss, thereby avoiding drought stress [7]. Mechano-stimulation was reported to increase cold tolerance in beans, tomato, and maize through maintenance of higher Photosystem II (PSII) efficiency and accumulation of higher levels of soluble sugars [8]. It was also suggested that similar defense mechanisms are operated in cold acclimation and mechano-stimulation, resulting in similar morphological and developmental changes [8]. Recently, analysis of transcript profiles indicated various defense response genes were induced by mechano-stimulation, and were related to cold stress response, including general stress response (GSR), rapid stress response (RSR), and rapid wound response (RWR) [9]. In addition, it has been proposed that mechanical disruption of the cell wall may induce stress signaling [10]. Cold stress is perceived by the plant through detection of changes in membrane fluidity and protein conformation. Secondary messengers such as Ca^2+^ and ROS are implicated in the initial signaling cascades in response to cold stress [1].

Many studies reported changes in ROS levels following mechano-stimulation [11, 12]. For instance, mechano-stimulation induced a significant increase in ROS levels in *Mesembryanthemum crystallinum* leaves [12]. Furthermore, proteomic studies have shown that plants transiently produce superoxide and H_2_O_2_, which might play critical roles in signal transduction during early wound response [13]. Mechano-stimulation induced increased expression of cytosolic H_2_O_2_-detoxifying enzyme, ascorbate peroxidase 2 (APX2) [14]. This increase in APX2 was independent of other mechanical wounding signals such as jasmonic acid (JA) or abscisic acid (ABA) [15]. It has also been suggested that NADPH-dependent H_2_O_2_ signals contribute to the activation of specific mechano-stimulated signals which are not activated by the JA or ABA [15]. The cellular steady-state level of ROS is tightly regulated by a complex network involving Ca^2+^, protein phosphorylation, and ROS-scavenging/producing enzymes during wound response [15]. In addition, mechanical wounding has been found to induce a burst of superoxide and apoplastic peroxidase with both oxidative and peroxidative activities [15, 16].

In this study, mechano-stimulation was applied to two contrasting winter wheat cultivars that differed in cold tolerance at different growth stages in order to investigate the effects of mechano-stimulation on the performance of the chloroplastic antioxidant system and changes of the proteome under late spring low temperature stress. The results obtained in this study may provide deeper insights into the roles of mechano-stimulated modifications within chloroplast antioxidant systems and proteome in cold tolerance in wheat. This information will be helpful for exploring novel approaches to mitigate the impacts of low temperatures which occur during critical developmental stages in wheat plants.

## Methods

### Plant materials

This experiment was carried out at Lianyungang Experimental Station of Nanjing Agricultural University (119°32′E, 34°30′N) during the wheat growing season in 2011–2012. The soil is a clay, contains 11.4 g kg^−1^ organic matter, 1.1 g kg^−1^ total N, 79.8 mg kg^−1^ available N, 32.4 mg kg^−1^ Olsen-P, and 132.4 mg kg^−1^available K. Before sowing, 120 kg N ha^−1^, 60 kg P_2_O_5_ ha^−1^ and 120 kg K_2_O ha^−1^ were applied as basal fertilizer and a further 120 kg N ha^−1^ was used as a topdressing after jointing to avoid the potential impacts on stress treatments. Two winter wheat cultivars differing in cold tolerance but having close genetic backgrounds (Jimai 17 displays similar morphology and is related with Yannong 19 in pedigree), Yannong19 (YN19, cold tolerant) and Lianmai6 (LM6, cold susceptible, parents: YN19// Jimai 17/Zheng9023) were used in this experiment. The sowing date was 14 October 2011, with a seedling density of 160 m^−2^ and a row space of 0.25 m. The jointing date was confirmed through spike development checked with a Dino-Lite digital microscope (AM411 Version 1.4.1; Vidy Precision Equipment Co. Ltd, Wuxi, China).

### Mechano-stimulation and cold treatments

To investigate the effects of mechano-stimulation applied at different stages on seedling performance under cold stress, four treatments were imposed: P_1_L, the early priming of mechano-stimulation for plants was applied at the Zadoks growth stage 26 (25 March 2012) and then subjected to a 4-day cold event at the Zadoks growth 31 (jointing stage, 8–12 April 2012); P_2_L, the later mechano-stimulation for plants was carried out 6 days before the cold event (2 April 2012); CL, the cold stress at jointing without early mechano-stimulation; CC, the normal temperature control. Mechano-stimulation was carried out using a cylinder roller with weight of 150 kg and diameter of 40 cm. The roller was rolled over the wheat plants with a pressure of 7000 N · m^−2^ at 9:00–9:30 am, which resulted in less than 20 % of the leaf area being damaged at jointing A 4-day cold stress was applied using four temperature control systems operated in the open top chamber condition. Air was cooled by a compressor, and then the cooled air was driven by an air blower to the field through ducting [16]. During cold treatment, plots were surrounded by 180-cm-high plastic film. All tubes were removed just after cooling treatment to avoid shading. Six temperature and humidity sensors were used to record the real-time data in each plot. The mean temperature in the cold treatment was 5.60 °C lower than the normal temperature control. The mean temperature at night was −1.14 °C, and the lowest temperature recorded during the cold treatment was −4.97 °C (detailed temperature data are shown in Additional file 1: Figure S1). The experiment had a split-plot design with temperature treatment as the main plot and wheat cultivar as the subplot, with three replicates for each treatment. The size of each plot was 3 m × 4 m.

### Chl a fluorescence transient

The fast chlorophyll a fluorescence induction curve was measured using a Plant Efficiency Analyzer (Pocket-PEA; Hansatech, Norfolk, UK) [17]. Before measuring, plants were dark adapted for 0.5 h. The collected data were processed by the program PEA Plus 1.04, and Biolyzer 3.0 software (Bioenergetics Lab., Geneva, Switzerland, http://www.fluoromatics.com/biolyzer_software-1.php) was used to calculate the fast chlorophyll a fluorescence induction (OJIP) test parameters.

### Chloroplast extraction and enzyme activity analysis

Chloroplasts were isolated and purified from the latest fully expanded leaves following our previous method with a few modifications [16]. Leaf samples (6 g) were ground in 30 ml of extraction buffer (0.45 M sucrose, 15 mM 3-(*N*-morpholino) propanesulfonic acid (MOPS), 1.5 mM ethylene glycol tetraacetic acid (EGTA), 0.6 % polyvinylpyrro-lidone (PVP), 0.2 % bovine serum albumin (BSA), 0.2 mM phenylmethylsulphonyl fluoride (PMSF) and 10 mM dithiothreitol (DTT)). The homogenate was filtered through eight layers of gauze, and the filtrate was then centrifuged at 2 000 × *g* for 5 min. The sediment was resuspended with sorbitol resuspension medium (SRM, 0.33 M sorbitol in 50 mM 4-(2-hydroxyethyl)-1-piperazineethanesulfonic acid (HEPES)), and then layered on the top of a 7-ml layered system (35 %, 80 % Percoll) for step gradients. The chloroplasts were collected and washed with 2 ml SRM followed by centrifugation at 1100 × *g* for 10 min. Finally, the intact chloroplasts were maintained in 2 ml SRM at −4 °C.

Following the methods of Zheng et al. [18], H_2_O_2_ concentration was measured by monitoring the absorbance of titanium peroxide complex at 410 nm, and the release rate of O_2_^−^ was determined at an absorbance at 530 nm. APX (EC 1.11.1.11) activity was determined by monitoring the decrease in absorbance at 290 nm, the activity of SOD (EC 1.15.1.1) was measured by monitoring the inhibition of photochemical reduction of nitroblue tetrazolium (NBT), and GPX (EC 1.11.1.7) activity was calculated by monitoring the increase in absorbance at 470 nm due to the oxidation of guaiacol. GR (EC 1.6.4.2) activity was determined by the oxidation of NADPH at 340 nm, and CAT (EC 1.11.1.6) activity was measured following the method of Tan et al. [19]. DHAR (EC 1.8.5.1) was assayed by monitoring changes in absorbance at 265 nm after the addition of ascorbate oxidase as described by Miyake and Asada [20]. The activities of Ca^2+^-ATPase and Mg^2+^-ATPase in the chloroplasts suspension were measured following the method of Zheng et al. [18].

### Rubisco activity

Leaf samples (0.2 g) were ground in 40 ml of extraction buffer (50 mM Tris-HCl, 1 mM EDTA, 1 mM MgCl_2_, 10 % PVP and 10 mM β-mercaptoethanol), and then centrifuged at 15 000 × *g* for 15 min. The supernatant was gently collected to measure Rubisco activity. The activity of Rubisco (EC 4.1.1.39) before (initial activity) and after (total activity) active site carbamylation was assayed using a spectrophotometric procedure coupled to NADH oxidation [21]. Rubisco activation was estimated as the percentage ratio of initial to total activities for each sample.

### Protein extraction and 2-DE procedure

The extraction of protein in the latest fully expanded leave for 2 DE was performed following the trichloroacetic acid (TCA) acetone precipitation method described by Ding et al. [22].

Immobiline DryStrip gels (117 cm length: Bio-Rad) were used for first dimension isoelectrofocusing (IEF) at pH 4 to 7. Rehydration and focus were performed using PROTEAN IEF apparatus (Bio-Rad) at 50 μA per strip at 20 °C, using the following programme: 12 h of rehydration at 50 V in rehydration buffer (7 M urea, 2 M thiourea, 4 % (w/v) CHAPS, 0.5 % (v/v) IPG buffer, 10 mM DTT, and 0.1 % bromophenol blue), 1 h at 500 V, 1 h at 1 000 V, 2 h at 8 000 V, and 85 000 V · hours at 8 000 V. After dimension isoelectrofocusing, strips were equilibrated for 15 min in SDS equilibration buffer solution (6 M urea, 37.5 mM Tris-HCl (pH 6.8), 20 % (v/v) glycerol, 2 % (w/v) SDS, and 1 % (w/v) DTT), followed by equilibration with a buffer containing 135 mM iodoacetamide for 15 min. After equilibration, proteins were distributed in the second dimension (SDS-PAGE) using 10 % polyacrylamide gels (250 × 200 × 1 mm), and the gels were stained with silver nitrate solution.

### Image analysis, protein identification, and functional annotation

The gels were scanned using a VersaDoc4000 image system (Bio-Rad) and the images were analysed with PDQUEST 8.0 software (Bio-Rad, USA). There were three biological replicates per treatment with at least three gels for each biological replicate. Only spots with a variation rate of ±0.5 in the three replicates were considered for further analysis. Stained protein spots were excised manually from the gels, in-gel digested with trypsin, and analysed using a MALDI-TOF/TOF mass spectrometer (ABI 4800). The MASCOT database search engine (http://matrixscience.com) was used to search for peptide mass lists from the obtained spectra against the NCBI database. The mass error tolerance was set to 80 ppm, and the score threshold was above or equal to 110.

### RNA extraction and qRT-PCR for gene expression analysis

RNA was extracted from wheat leaves using Trizol according to the manufacturer’s instructions. The gene-specific primers were constructed using the Primer 3 programme, on the basis of wheat gene sequences in the GenBank (http://www.ncbi.nlm.nih.gov/) [23]. The following primers were used for amplification: *Cu/Zn SOD*, 5′-CGCTCAGAGCCTCCTCTTT-3′ and 5′-CTCCTGGGGTGGAGACAAT-3′; *Fe SOD*, 5′-GAAGCTTGAGGTGGCACA-3′ and 5′-TAAGCATGCTCCCACAAGTC-3′; *CAT*, 5′-CCATGAGATCAAGGCCATCT-3′ and 5′-ATCTTACATGCTCGGCTTGG-3′; *tAPX*, 5′-G CAGCTGCTGAAGGAGAAGT-3′ and 5′-CACTGGGGCCACTCACTAAT-3′; *β-actin*, 5'- GCTCGACTCTGGTGATGGTG-3' and 5'- AGCAAGGTCCAAACGAAGGA-3'. The qPCR analysis was performed using the TaKaRa® SYBR Premix Ex Taq™ II on an ABI PRISM 7300 Sequence Detection System (ABI, Foster, CA, USA). The PCR conditions consisted of denaturation at 95 °C for 3 min, followed by 40 cycles of denaturation at 95 °C for 15 s, annealing at 54 °C for 20 s, and extension at 72 °C for 18 s. To minimize sample variations, *β-actin* was used as the reference gene. Each extraction and qRT-PCR was replicated three times. The quantification of mRNA levels was based on the relative quantification method (2^-ΔΔCt^) [24].

### Statistical analysis

All data were subjected to the two-way ANOVA using the SigmaSATA (Systat Software Inc., CA, USA). The Duncan’s multiple range test was used to check the significance of difference between treatments. In 2-DE analysis, the difference of expression level at the given protein spots between treatments and the control (CC) for each cultivar was calculated and converted to a color scale by PageMan software (http://mapman.mpimp-golm.mpg.de/pageman/).

## Results

### Chl a fluorescence transient

**Fig. 1:**
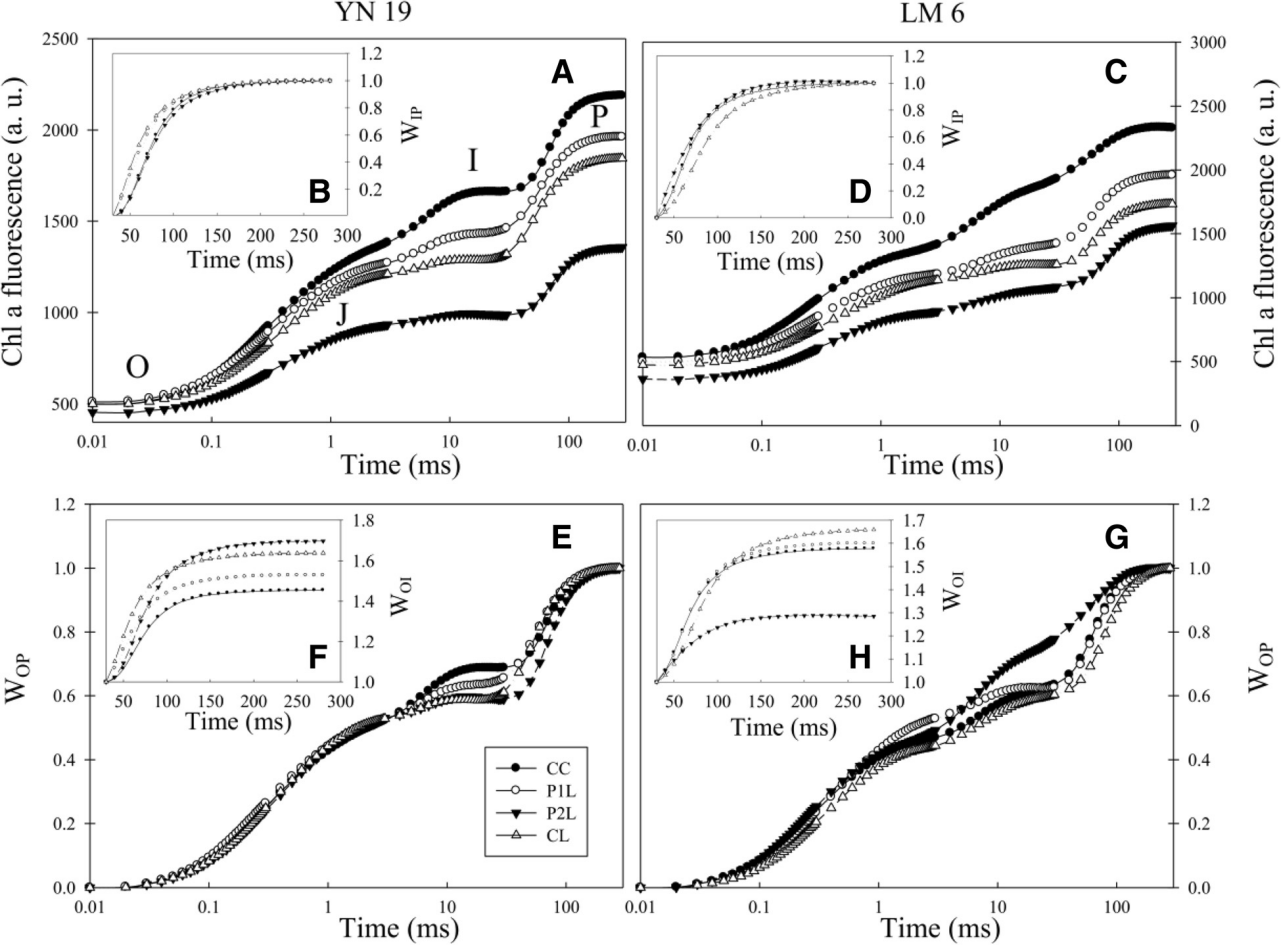
Effects of mechano-stimulation on chlorophyll a fluorescence transient of dark adapted leaves (the latest fully expanded leaves) in winter wheat exposed to cold stress at jointing. (**a & c**) fluorescence intensity on logarithmic time scale; **b & d** W_IP_ = (F_t_-F_I_)/(F_P_-F_I_), ratio of variable fluorescence F_t_-F_I_ to the amplitude F_P_-F_I_; (**e & g**) W_OP_ = (F_t_-F_O_)/(F_P_-F_O_), ratio of variable fluorescence F_t_-F_O_ to the amplitude F_P_-F_O_; (**f & h**) W_OI_ = (F_t_-F_O_)/(F_I_-F_O_), ratio of variable fluorescence F_t_-F_O_ to the amplitude F_I_-F_O_

The increase in leaf fluorescence transients observed in CC treatment showed a typical OJIP shape in YN19 and LM6 (Fig. 1a, c). However, P_1_L (early mechano-stimulation + cold stress), P_2_L, (later mechano-stimulation + cold stress) and CL (non-mechano-stimulation + cold stress) showed repressed fluorescence transients in these two cultivars, particularly at step I (30 ms) and P. The main changes of fluorescence data were normalized between step I (30 ms) and P (300 ms) and presented as relative variable fluorescence W_IP_ (Fig. 1b, d). Obvious changes in W_IP_ during the fast rise period were observed under P_2_L and CL in YN19, while under P_1_L, P_2_L, and CL in LM6, compared with CC. W_OP_ (Fig. 1e, g) and W_OI_ (Fig. 1f, h) showed relatively variable fluorescence from O to step P (300 ms) and from O to I (30 ms). A significant decrease in W_OP_ at step I was found in P_1_L, P_2_L, and CL in YN19, while W_OP_ was increased remarkably by P_2_L at step I in LM6. Significant changes in W_OI_ were found among P_1_L, P_2_L, and CL treatments in YN19, which were related to the reductions between PSI and reduced NADP^+^. However, with the exception of the P_2_L treatment, W_OI_ was only slightly affected in LM6.

### Rubisco activities and activation

**Fig. 2:**
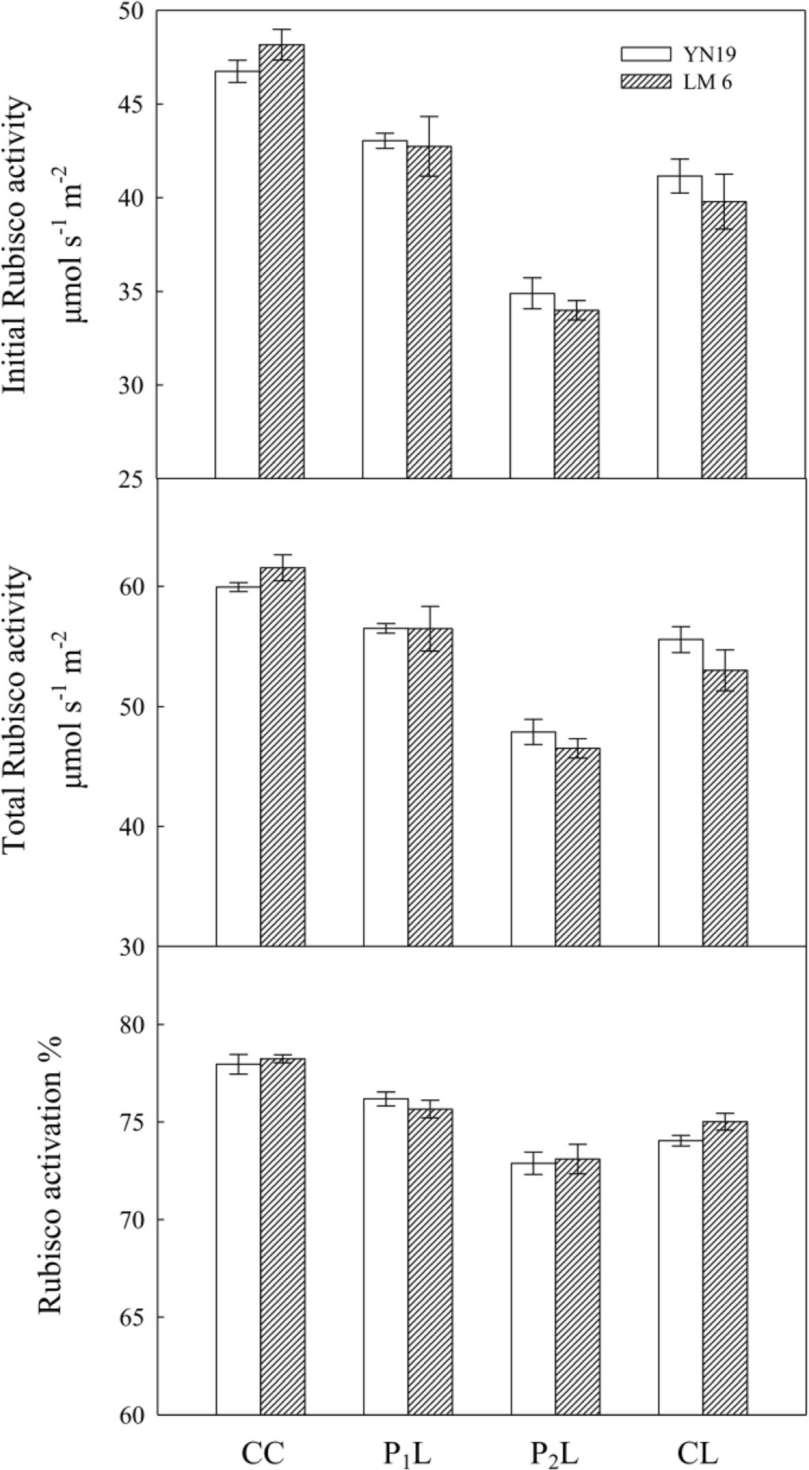
Effects of mechano-stimulation on initial and total Rubisco activities and activation in the latest fully expanded leaves in winter wheat exposed to cold stress at jointing

Initial and total Rubisco activities and Rubisco activation in the latest fully expanded leaves were significantly decreased with CL, compared with CC in YN19 and LM6 (Fig. 2, *P* < 0.001). Both traits were slightly and marginally significantly increased by P_1_L (*P* = 0.077), whereas they were depressed by P_2_L compared with CL (*P* < 0.001). Rubisco activation in P_1_L was relatively higher than in CL, but was still lower than in CC for both cultivars (*P* < 0.001). In addition, no difference in Rubisco activation was found between P_2_L and CL.

### ROS production, activities of antioxidant enzymes, and expressions of their encoding genes in chloroplasts

**Fig. 3:**
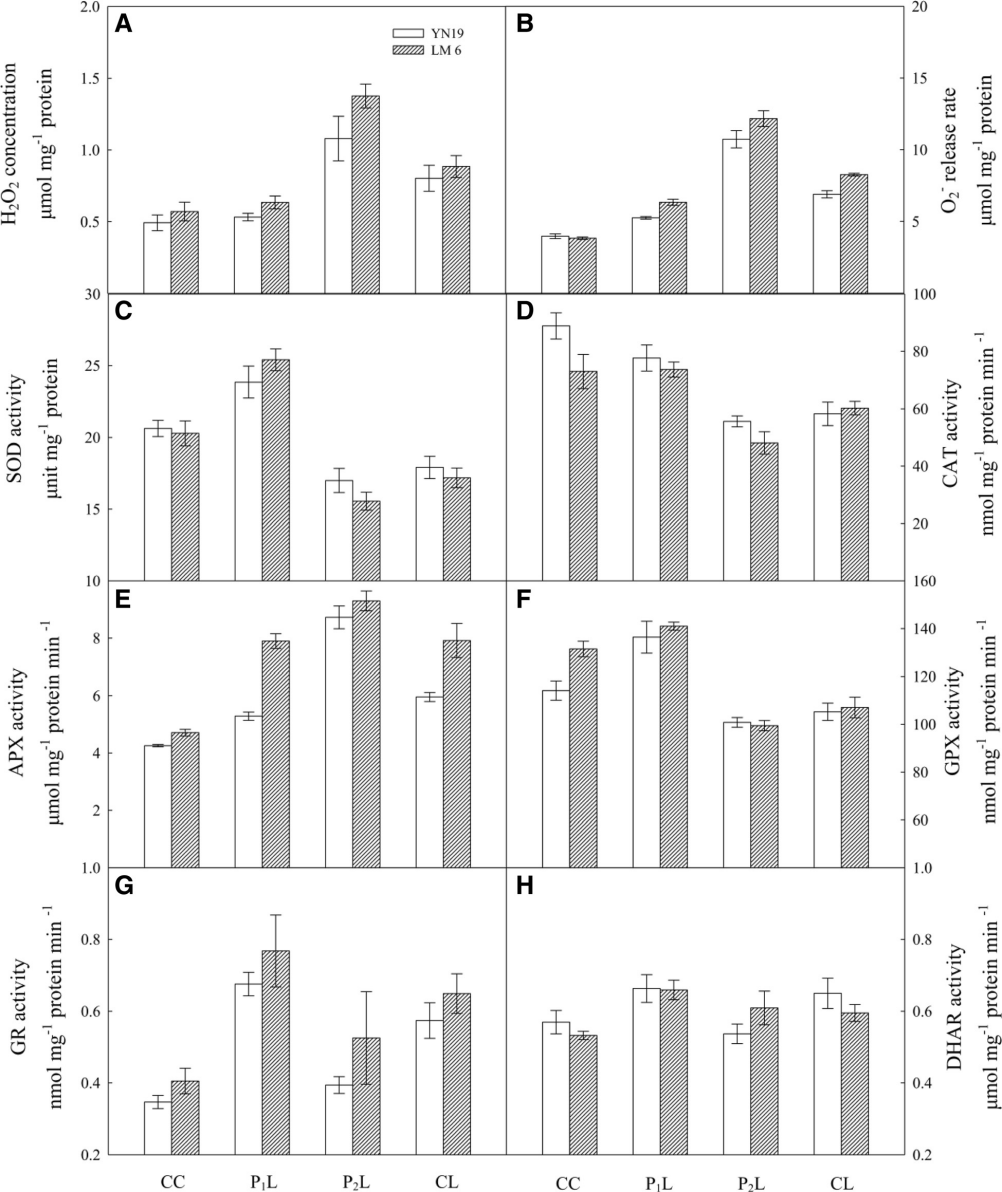
Effects of mechano-stimulation on reactive oxygen species and antioxidant enzyme system in chloroplasts in the latest fully expanded leaves in winter wheat exposed to cold stress at jointing. **a** H_2_O_2_, hydrogen peroxide; **b** O_2_^−^, superoxide anion radical; **c** SOD, superoxide dismutase; **d** CAT, catalase; **e** APX, ascorbate peroxidase; **f** GPX, glutathione peroxidase; **g** GR, glutathione reductase; **h** DHAR, monodehydroascorbate reductase

**Fig. 4:**
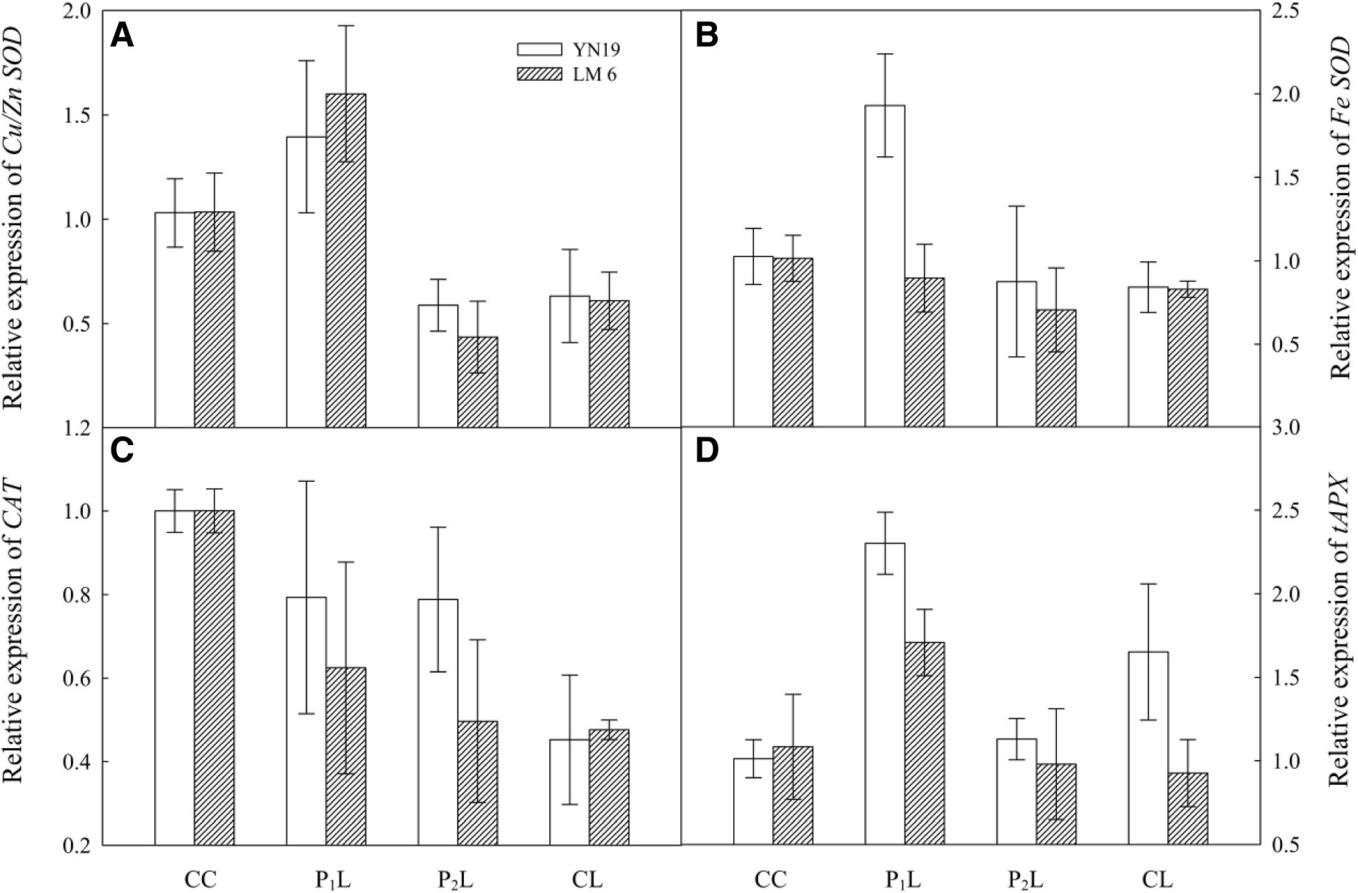
Effects of mechano-stimulation on relative transcript abundance of *Cu/Zn SOD* (**a**), *Fe SOD* (**b**), *CAT* (**c**) and *thylakoid-bound APX* (*tAPX*, **d**) in the latest fully expanded leaves in winter wheat exposed to cold stress at jointing

In YN19, CL increased the concentration of H_2_O_2_ in chloroplasts in the latest fully expanded leaves by 63 % as compared with CC, whereas P_2_L showed a 34 % increment compared with CL (Fig. 3a). However, no difference was observed between CC and P_1_L. A similar pattern was observed in LM6. The highest rate of O_2_^−^ release was found in P_2_L, followed by CL and P_1_L, whereas that in CC was lowest in both cultivars (Fig. 3b, *P* < 0.001). SOD activity in chloroplasts was increased by 16 % and 25 % with P_1_L in YN19 and LM6, respectively. Compared with CC, P_1_L increased while P_2_L and CL significantly decreased chloroplastic SOD activity in the two cultivars (Fig. 3c). In addition, in both cultivars, expression of *Cu/Zn SOD* was up-regulated by P_1_L compared with CL (Fig. 4a), whereas an up-regulation of *Fe SOD* due to P_1_L was only observed in YN19 (Fig. 4b). For both cultivars, CAT activity was lower in CL than in CC, whereas it was higher in P_1_L than in CL (Fig. 3d). In LM6, P_2_L decreased CAT activity by 20 % compared with CL, whereas no significant difference was found in YN19 (*P* = 0.112). An increase in the expression of *CAT* was found in P_1_L and P_2_L compared with CL in YN19; however, the difference was not statistically significant (Fig. 4c). In both cultivars, the combination of mechano-stimulation and low temperature (P_1_L and P_2_L) and CL enhanced APX activity compared with CC. In particular APX activity in P_2_L was significantly higher than in CL (Fig. 3e). Further, the same trend was found in *thylakoid-bound APX* (*tAPX*) expressions in YN19, whereas in LM6, a significant up-regulation of *tAPX* was only observed in P_1_L (Fig. 4d). In both cultivars, GPX activity was enhanced with P_1_L, but depressed with P_2_L and CL (no significant difference between P_2_L and CL) (Fig. 3f). In both cultivars, P_1_L and CL resulted in a significant increase in GPX activity, compared with CC (*P* < 0.001), whereas P_2_L slightly increased GPX activity (Fig. 3g, *P* = 0.105). Low temperature significantly enhanced DHAR activity in both cultivars (*P* < 0.001); however, P_1_L and P_2_L had opposite effects on DHAR activity in the two cultivars—namely, P_2_L decreased DHAR activity compared to CL in YN19, but increased activity in LM6; P_1_L increased DHAR activity in LM6, whereas no difference between P_1_L and CL was found in YN19 (Fig. 3h). Thus, P_1_L and P_2_L showed opposite patterns in the concentration of H_2_O_2_ in chloroplasts, O_2_^−^ release rate and most of the antioxidant enzyme activities, but the APX activity showed a similar trend in P_1_L and P_2_L.

### ATPase activities in chloroplasts

**Fig. 5:**
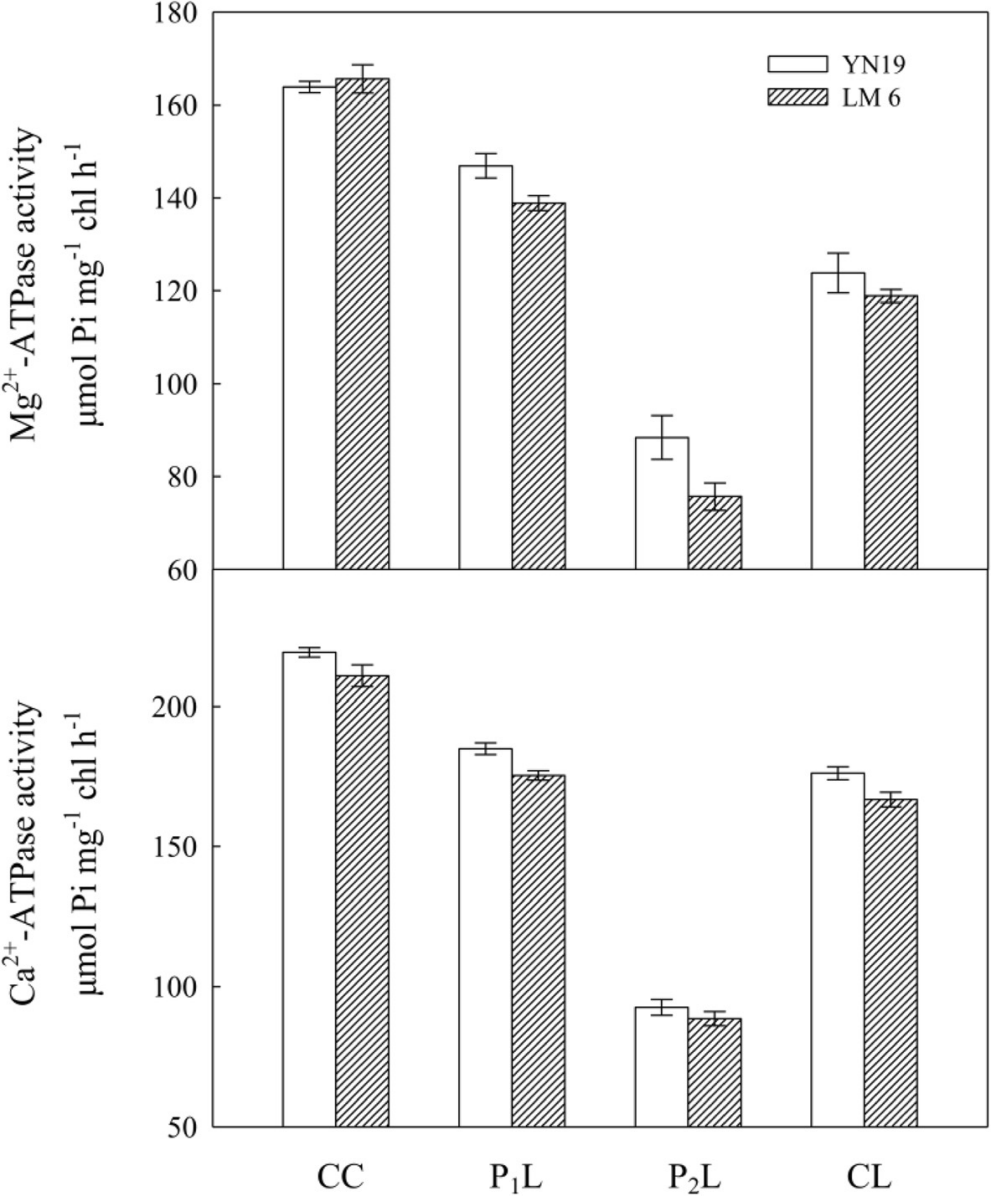
Effects of mechano-stimulation on activities of Mg^2+^-ATPase and Ca^2+^-ATPase in chloroplasts in the latest fully expanded leaves in winter wheat exposed to cold stress at jointing

In YN19, the Activities of both Mg^2+^-ATPase and Ca^2+^-ATPase were significantly decreased by CL as compared with CC (Fig. 5, *P* < 0.001). However, both ATPase activities were increased by P_1_L, whereas they decreased by P_2_L. The activities of Mg^2+^-ATPase and Ca^2+^-ATPase in LM6 in response to different treatments were similar to those in YN19.

### Proteomics

**Fig. 6:**
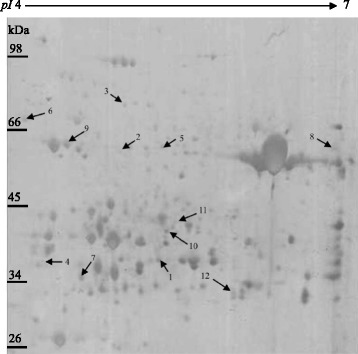
Reference 2-DE gel of proteins in wheat leaves under combination of mechano-stimulation and jointing cold stress. Differentially expressed protein spots in stress treatments (CL, P_1_L and P_2_L) compared with CC for each cultivar were indicated with arrows and listed in Table 1

**Fig. 7:**
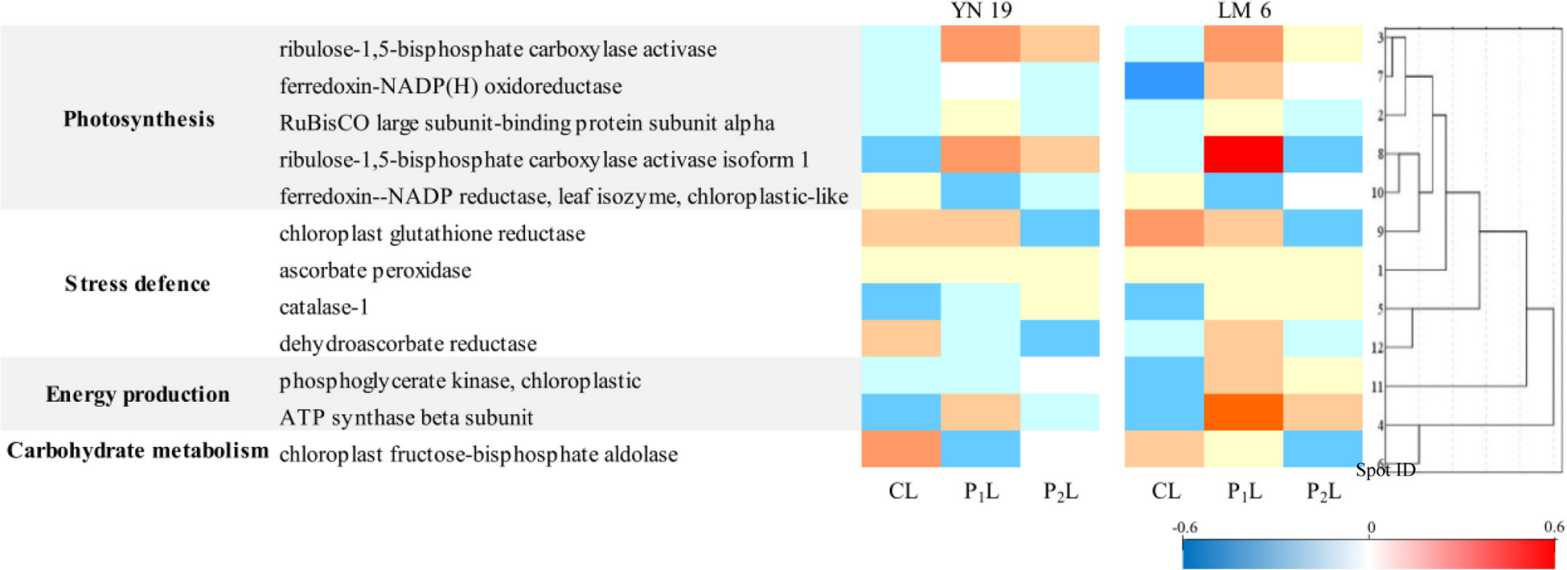
Relative expression ratio of altered proteins in wheat leaves affected by combination of mechano-stimulation and jointing cold stress. The difference of expression level at the given protein spots between CL and CC, P_1_L (or P_2_L) and CL was log-normalized and converted to a color scale. It was reorganized after analysis with the PageMan software (http://mapman.mpimp-golm.mpg.de/pageman/). Up-regulation and down- regulation were indicated in increasing *red* and *blue*, respectively. The missing proteins were indicated in *white*

The reference 2-DE gel of proteins in wheat leaves affected by combination of mechano-stimulation and cold stress is shown in Fig. 6. More than 600 protein spots were detected in each gel. To demonstrate the proteomic response of the photosynthetic apparatus to mechano-stimulation and cold stress, variation in the expression of 12 protein spots related to photosynthesis, energy production, stress defense in chloroplasts is specifically shown in Fig. 7. The differentially expressed protein spots were identified by mass spectrometry (MS, Table 1). In the cluster related to photosynthesis, five protein spots, including enzymes involved in the Calvin cycle and Rubisco protein subunit—ferredoxin-NADP(H) oxidoreductase (spot 10), ribulose-1, 5-bisphosphate carboxylase activase (spot 11) and the Rubisco large subunit-binding protein subunit alpha (6)—were up-regulated by P_1_L in both cultivars; the exception being spot 10, which was missing in P_1_L in YN19. CL induced up-regulation in chloroplastic glutathione reductase (spot 5) and ascorbate peroxidase (spot 7) in both cultivars, whereas the expression of catalase-1 (spot 8) was down-regulated by CL. These proteins were, however, all up-regulated by P_1_L in both cultivars, except for catalase-1 in YN19. In addition, in both cultivars, the expression of ATP synthase β subunit (spot 9) was depressed by CL compared with CC, but was increased by P_1_L compared with CL. Interestingly, the chloroplastic fructose-bisphosphate aldolase (spot 2) was up-regulated by CL in the two cultivars, whereas under P_1_L, it was decreased in YN19 but increased in LM6. Proteomic analyses revealed that the oxidative stress defense, ATP synthesis, and photosynthesis-related proteins were similarly modulated by the mechano-stimulation and the cold stress.

**Table 1:** Identification of differentially expressed proteins in wheat leaves affected by combination of mechano-stimulation and jointing cold stress through MALDI-TOF/TOF

Spot ID	Protein name	GI accession no.	Theor. Mr (kDa)/pI	Score	SC (%)	Taxonomy
1	ferredoxin-NADP reductase, leaf isozyme, chloroplastic-like	357110920	40.81/6.72	103	5	*Brachypodium distachyon*
2	chloroplast fructose-bisphosphate aldolase	223018643	42.22/5.94	800	30	*Triticum aestivum*
3	Phosphoglycerate kinase, chloroplastic	129915	49.98/6.58	323	10	*Triticum aestivum*
4	ribulose-1,5-bisphosphate carboxylase activase isoform 1	167096	47.37/8.62	549	45	*Hordeum vulgare subsp*
5	chloroplast glutathione reductase	148250114	50.87/6.17	309	10	*Dasypyrum villosum*
6	RuBisCO large subunit-binding protein subunit alpha, chloroplastic precursor	134102	57.66/4.83	841	26	*Triticum aestivum*
7	ascorbate peroxidase	15808779	27.96/5.10	471	27	*Hordeum vulgare subsp. Vulgare*
8	catalase-1	2493543	57.00/6.52	512	13	*Triticum aestivum*
9	ATP synthase beta subunit	110915710	53.02/5.17	326	17	*Vulpia microstachys*
10	ferredoxin-NADP(H) oxidoreductase	20302473	40.49/6.92	739	34	*Triticum aestivum*
11	ribulose-1,5-bisphosphate carboxylase activase	37783283	22.49/4.98	475	34	*Triticum aestivum*
12	dehydroascorbate reductase	28192421	23.46/5.88	118	22	*Triticum aestivum*

Spot ID are named according to Fig. 6. GI refers to accession number. NCBI refers to database accession number. Mr/pI refers to molecular weight and isoelectric point of identified protein. Score refers to Mascot protein score. SC refers to Sequence Coverage

## Discussion

It is well known that ROS production is a universal response to mechanical wounding in various plants [11]. The defense system can also be activated to alleviate ROS-induced oxidative stress and repair the damaged tissues [25]. Furthermore, many of the genes encoding enzymes involved in ROS metabolism are regulated by mechanical wounding [26]. Here, the leaf chloroplastic H_2_O_2_ concentration in P_1_L plants was very close to that in CC plants. In contrast, P_2_L plants have a significantly higher H_2_O_2_ concentration than CC plants (Fig. 3). This difference is related to the efficient ROS scavenging capacity of the antioxidant enzyme systems, particularly the water-water cycle in chloroplasts, which mainly includes SOD and APX [27]. The scavenging capacity of SOD and CAT activated by the mechano-stimulation in P_1_L had a significant inhibitory effect on the oxidative burst under low temperature stress. Further analysis revealed that the enhanced activities of SOD and CAT could be largely explained by the up-regulated expression of *Cu/Zn SOD*, *Fe SOD*, and *CAT* in P_1_L (Fig. 7). Wound-induced activation of H_2_O_2_-detoxifying enzymes has previously been demonstrated using proteomic tools [26]. Our proteome analysis showed that the expression levels of ascorbate peroxidase and catalase-1 were paralleled by the activities of APX and CAT, respectively, under different treatments (Fig. 7). However, in YN19, the activity of APX in P_1_L was decreased compared to CL, whereas no significant difference was found in LM6. The qPCR analysis also showed that the *tAPX* expression was only slightly affected by P1L in YN19, but it was increased by P_1_L in LM6. APX activity under the combination of mechano-stimulation and cold stress was only partly consistent with that previously reported. It has been reported that expression of ascorbate peroxidase 2 (APX2) is involved in modulation of cellular H_2_O_2_ levels in response to wounding [15, 26, 27]. The increase in APX activity in P_2_L and no increase in P_1_L suggested that APX may not play vitally important roles in the mechano-induced cold tolerance in wheat.

Increasing evidence supports the multi-signaling functions of H_2_O_2_ in response to abiotic stresses in higher plants [11]. Here, under low temperature, for both texted cultivars the H_2_O_2_ concentration in P_1_L was very close to the normal level in CC. However, the release rate of O_2_^−^ in P_1_L was significantly higher than in CC. It was suggested that activated antioxidative enzymes, such as SOD and CAT induced by mechano-stimulation, modify the H_2_O_2_ concentration to an appropriate level as a signal molecule, which prevents H_2_O_2_-induced damage to plant tissues [11]. In addition, modified GPX and GR activities have also been shown to be related to the down-regulation of H_2_O_2_ levels in chloroplasts [28]. Here, the increased activities of GPX and GR did favour the relatively low level of H_2_O_2_ in P_1_L (Fig. 3). Although the concentrations of AsA and GSH are only in the millimolar range in plant tissues, the AsA-GSH cycle plays a very important role in neutralizing H_2_O_2_ released by disproportionation of O_2_^−^ [28]. As key members in the AsA-GSH cycle, the altered expression of glutathione reductase (GR)- and dehydroascorbate reductase (DHAR)-related proteins were found via proteome analysis in the present study; chloroplastic GR was increased in P_1_L in both cultivars, whereas DHAR was enhanced in P_1_L only in LM6 compared to CL (Fig. 7). The changes in expression of these enzymes are in accordance with their activities in chloroplasts. Thus, we suggest that the AsA-GSH cycle is involved in mechano-stimulated cold tolerance in winter wheat.

Hardening with a previous abiotic stress endows plant with higher tolerance to recurring stresses [29]. For example, pre-anthesis heat hardening (or pre-treatment) can partially protect wheat plants from photosynthetic inhibition and oxidative damage under post-anthesis high-temperature stress, which is attributed to the modified expressions of photosynthesis-responsive and antioxidant enzyme-related genes [30]. Furthermore, many studies have shown that the mechanism underlying hardening includes the accumulation of soluble sugars, reduction of photosynthetic apparatus [30], scavenging of reactive oxygen species (ROS) [30], accumulation of osmoprotective proteins (dehydrins) [31], and other compatible solutes such as proline and betaína [31]. It is well known that cold acclimation reduces frost damage, and that this phenomenon involves a mechanism similar to that of drought acclimation [32]. Mechano-stimulation may induce many types of cold response proteins and genes [8]. In addition to the antioxidant system activated by mechano-stimulation, shown in the present study, many types of proteins related to photosynthesis, energy production, and C metabolism were modified by mechano-stimulation (Fig. 7). With respect to photosynthetic C assimilation, ribulose-1, 5-biophosphate carboxylase activase and its isoform 1 had a higher level of expression in P_1_L, but a relatively low level in CL, in the two tested cultivars. Ribulose-1, 5-biophosphate carboxylase activase and Rubisco large subunit-binding protein have been shown to play a critical role in the activation of Rubisco [33]. Carbonic anhydrase enhances the CO_2_ concentration in chloroplasts, which improves the carboxylation rate of Rubisco enzyme [21]. Our proteomic data also showed a higher abundance of Rubisco large subunit-binding protein subunit α in P_1_L than that in CL in both cultivars. This implies increases in the Rubisco activation state and carboxylation rate induced in the early mechano-stimulated plants under low temperature stress (Fig. 2). The unaltered expression of ferredoxin-NADP(H) oxidoreductase and down-regulation of ferredoxin-NADP reductase, leaf isozyme, and chloroplastic-like protein in P_1_L observed in the present study, resulted in an increased level of NADPH-dependent H_2_O_2_ as compared with CL. It has been suggested that increased NADPH-dependent H_2_O_2_ is required for the activation of systemic wound responses [11]. The mechano-stimulation induced H_2_O_2_ production is also involved in plant defence responses against invading pathogens [34]. It was reported that ROS can control Ca^2+^-permeable channel activity to regulate the intracellular Ca^2+^ level [35]. The changes of ROS and Ca^2+^ following mechano-stimuli were implicated in the induction of defense genes in response to fungal pathogens [34, 36].

Photosynthetic electron transport generates energy (ATP) and reducing power (NADPH) to support carbon reduction and photorespiratory carbon oxidation in the dark reaction in photosynthesis and plays a key role in the maintenance of optimum photosynthetic rate and ensuring effective energy flow for growth [37]. To further investigate the interactive effects of mechano-stimulation and cold stress on the process of photosynthetic electron transport, transient fluorescence kinetics were analysed (Fig. 1). The I-P phase of the transient fluorescence kinetics revealed changes in the electron flux from PQH_2_ to the final electron acceptor and the size of the final electron acceptor pool of PS I [38]. The present study showed no significant effects of the combination of mechano-stimulation and cold stress on the electron flux from PQH_2_ to the final electron acceptor. However, the O-I part of the kinetics was affected by cold stress and mechano-stimulation, which reveals changes in the process involving exciton capture to PQ reduction [38]. In addition, the rise in fluorescence transient from O to P was faster in P_2_L in LM6, which indicates that the re-oxidation of Q_A_^−^ was inhibited by the combination of cold and later mechano-stimulation [39]. However, it was not markedly affected by cold stress alone. The ATPases in chloroplasts, Mg^2+^-ATPase and Ca^2+^-ATPase, play a key role in ATP formation [18]. In this study, the activities of these two functional enzymes in chloroplasts were enhanced by mechano-stimulation in response to cold stress, which might favor the ATP formation. Consistently, the proteome data showed that, in both cultivars tested, there was a higher abundance of ATP synthase β subunit in P_1_L.

## Conclusions

Early mechano-stimulation at the stage of at Zadoks growth stage 26 activated the antioxidant system and hence maintained the balance of reactive oxygen species, improved the electron transport and photosynthesis rate under cold stress applied at the jointing stage, whereas mechano-stimulation applied 6 days before the cold event induced an opposite effect, except for APX activity and ATPase activities in chloroplasts. Proteomic and transcriptional analysis revealed that the oxidative stress defense, ATP synthesis, and photosynthesis-related proteins and genes are up-regulated by the mechano-stimulation, which were involved in the responses of wheat plants to the cold stress.

## Additional file

Additional file 1: Figure S1. Temperature difference between low temperature treatment and the normal temperature control during the cold stress treatment at jointing. (DOCX 1175 kb)

## Abbreviations

ABA: Abscisic acid
APX: Ascorbate peroxidase
AsA: Ascorbate
ATP: Adenosine triphosphate
BSA: Bovine serum albumin
CAT: Catalase
CC: the normal temperature control
CL: The cold stress at jointing without early mechano-stimulation
DHAR: Dehydroascorbate reductase
DTT: Dithiothreitol
EGTA: Ethylene glycol tetraacetic acid
GPX: Glutathione peroxidase
GR: Glutathione reductase
GSR: General stress response
HEPES: 4-(2-hydroxyethyl)-1-piperazineethanesulfonic acid
H2O2: Hydrogen peroxide
IEF: Isoelectrofocusing
JA: Jasmonic acid
LEA-related COR protein: Late embryogenesis abundant protein-related cold-responsive protein
MOPS: 3-(N-morpholino) propanesulfonic acid
MS: Mass spectrometry
NADPH: Nicotinamide adenine dinucleotide phosphate
NBT: Nitroblue tetrazolium
O_2_: Singlet oxygen
P_1_L: The combined treatment of early priming of mechano-stimulation at the Zadoks growth stage 26 and a 4-day cold event at the jointing stage
P_2_L: The combined treatment of the later mechano-stimulation 6 days before the cold event and a 4-day cold event at the jointing stage
PMSF: Phenylmethylsulphonyl fluoride
PSII: Photosystem II
PVP: Polyvinylpyrro-lidone
ROS: Reactive oxygen species
RSR: Rapid stress response
RWR: Rapid wound response
SDS: Sodium dodecyl sulfate
SOD: Superoxide dismutase
SRM: Sorbitol resuspension medium
TCA: Trichloroacetic acid

## References

[1] Ruelland E, Vaultier MN, Zachowski A, Hurry V, Jean-Claude K, Delseny M (2009). Cold signalling and cold acclimation in plants. Advances in Botanical Research.

[2] Chen S, Yin C, Strasser RJ, Govindjee, Yang C, Qiang S (2012). Reactive oxygen species from chloroplasts contribute to 3-acetyl-5-isopropyltetramic acid-induced leaf necrosis of *Arabidopsis thaliana*. Plant Physiol Bioch.

[3] Rinalducci S, Egidi MG, Karimzadeh G, Jazii FR, Zolla L (2011). Proteomic analysis of a spring wheat cultivar in response to prolonged cold stress. Electrophoresis..

[4] Han Q, Kang G, Guo T (2013). Proteomic analysis of spring freeze-stress responsive proteins in leaves of bread wheat (*Triticum aestivum* L.). Plant Physiol Bioch.

[5] Shimmen T (2001). Electrical perception of “death message” in Chara: Involvement of turgor pressure. Plant Cell Physiol..

[6] Engelberth J, Contreras CF, Viswanathan S (2012). Transcriptional analysis of distant signaling induced by insect elicitors and mechanical wounding in *Zea mays*. PLoS ONE..

[7] Jaffe M, Forbes S (1993). Thigmomorphogenesis: the effect of mechanical perturbation on plants. Plant Growth Regul..

[8] Keller E, Steffen KL (1995). Increased chilling tolerance and altered carbon metabolism in tomato leaves following application of mechanical stress. Physiol Plantarum..

[9] Walley JW, Coughlan S, Hudson ME, Covington MF, Kaspi R, Gopalan B, Harmer SL, Dehesh K (2007). Mechanical stress induces biotic and abiotic stress responses via a novel cis-element. PLoS Genet..

[10] Vorwerk S, Somerville S, Somerville C (2004). The role of plant cell wall polysaccharide composition in disease resistance. Trends Plant Sci..

[11] Orozco-Cardenas M, Ryan CA (1999). Hydrogen peroxide is generated systemically in plant leaves by wounding and systemin via the octadecanoid pathway. P Natl Acad Sci USA.

[12] Ślesak I, Ślesak H, Libik M, Miszalski Z (2008). Antioxidant response system in the short-term post-wounding effect in *Mesembryanthemum crystallinum* leaves. J Plant Physiol..

[13] León J, Rojo E, Sánchez-Serrano JJ (2001). Wound signalling in plants. J Exp Bot..

[14] Chang C, Ball L, Fryer MJ, Baker NR, Karpinski S, Mullineaux PM (2004). Induction of *ASCORBATE PEROXIDASE 2* expression in wounded *Arabidopsis* leaves does not involve known wound-signalling pathways but is associated with changes in photosynthesis. Plant J..

[15] Suzuki N, Mittler R (2012). Reactive oxygen species-dependent wound responses in animals and plants. Free Radical Bio Med..

[16] Li X, Cai J, Liu F, Dai T, Cao W, Jiang D (2014). Cold priming drives the sub-cellular antioxidant systems to protect photosynthetic electron transport against subsequent low temperature stress in winter wheat. Plant Physiol Biochem..

[17] Strasser R, Tsimilli-Michael M, Srivastava A, Papageorgiou GC, Govindjee (2004). Analysis of the chlorophyll a fluorescence transient. Chlorophyll Fluorescence: A Signature of Photosynthesis.

[18] Zheng C, Jiang D, Liu F, Dai T, Jing Q, Cao W (2009). Effects of salt and waterlogging stresses and their combination on leaf photosynthesis, chloroplast ATP synthesis, and antioxidant capacity in wheat. Plant Sci..

[19] Tan W, Liu J, Dai T, Jing Q, Cao W, Jiang D (2008). Alterations in photosynthesis and antioxidant enzyme activity in winter wheat subjected to post-anthesis water-logging. Photosynthetica..

[20] Miyake C, Asada K (1992). Thylakoid-bound ascorbate peroxidase in spinach chloroplasts and photoreduction of its primary oxidation product monodehydroascorbate radicals in thylakoids. Plant Cell Physiol..

[21] Pérez P, Alonso A, Zita G, Morcuende R, Martínez-Carrasco R (2011). Down-regulation of Rubisco activity under combined increases of CO_2_ and temperature minimized by changes in Rubisco kcat in wheat. Plant Growth Regul..

[22] Ding C, You J, Liu Z, Rehmani MIA, Wang S, Li G, Wang Q, Ding Y (2011). Proteomic analysis of low nitrogen stress-responsive proteins in roots of rice. Plant Mol Biol Rep..

[23] Baek KH, Skinner DZ (2003). Alteration of antioxidant enzyme gene expression during cold acclimation of near-isogenic wheat lines. Plant Sci..

[24] Livak KJ, Schmittgen TD (2001). Analysis of relative gene expression data using real-time quantitative PCR and the 2(−Delta Delta C(T)) Method. Methods..

[25] Minibayeva F, Kolesnikov O, Chasov A, Beckett RP, Lüthje S, Vylegzhanina N, Buck F, Böttger M (2009). Wound-induced apoplastic peroxidase activities: their roles in the production and detoxification of reactive oxygen species. Plant Cell Environ..

[26] Soares NC, Wojtkowska J, Jackson PA (2011). A proteomic analysis of the wound response in Medicago leaves reveals the early activation of a ROS-sensitive signal pathway. J Proteomics..

[27] Asada K (1999). The water-water cycle in chloroplasts: scavenging of active oxygens and dissipation of excess photons. Ann Rev Plant Biol..

[28] Keunen E, Peshev D, Vangronsveld J, Van den Ende W, Cuypers A (2013). Plant sugars are crucial players in the oxidative challenge during abiotic stress: Extending the traditional concept. Plant Cell Environ..

[29] Wang X, Wollenweber B, Jacobsen S, Liu F, Jiang D (2011). Pre-anthesis high-temperature acclimation alleviates damage to the flag leaf caused by post-anthesis heat stress in wheat. J Plant Physiol..

[30] Munné-Bosch S, Alegre L (2000). Changes in carotenoids, tocopherols and diterpenes during drought and recovery, and the biological significance of chlorophyll loss in Rosmarinus officinalis plants. Planta..

[31] Bohnert HJ (2000). What makes desiccation tolerable?. Genome Biol..

[32] Janska AA, Marsik P, Zelenkova S, Ovesna J (2010). Cold stress and acclimation - what is important for metabolic adjustment?. Plant Biol..

[33] Kang G, Li G, Xu W, Pen X, Han Q, Zhu Y, Guo T (2012). Proteomics reveals the effects ofsalicylic acid on growth and tolerance to subsequent drought stress in wheat. J. Proteome Res..

[34] Chehab EW, Eich E, Braam J (2009). Thigmomorphogenesis: a complex plant response to mechano-stimulation. J. Exp. Bot..

[35] Ma W, Smigel A, Tsai Y, Braam J, Berkowitz GA (2008). Innate immunity signaling: cytosolic Ca^2+^ elevation is linked to downstream nitric oxide generation through the action of calmodulin or a calmodulin-like protein. Plant Physiol..

[36] Walley JW, Coughlan S, Hudson ME, Covington MF, Kaspi R, Banu G, Harmer SL, Dehesh K (2007). Mechanical stress induces biotic and abiotic stress responses via a novel cis-element. PLoS Genet..

[37] Ye Z, Robakowski P, Suggett D (2013). A mechanistic model for the light response of photosynthetic electron transport rate based on light harvesting properties of photosynthetic pigment molecules. Planta..

[38] Yusuf MA, Kumara D, Rajwanshia R, Strasserb RJ, Tsimilli-Michaelb M, Govindjee X, Sarina NB (2010). Overexpression of γ-tocopherol methyl transferase gene in transgenic Brassica juncea plants alleviates abiotic stress: Physiological and chlorophyll a fluorescence measurements. BBA Bioenergetics.

[39] Chen S, Zhou F, Yin C, Strasser R, Yang C, Qiang S (2011). Application of fast chlorophyll a fluorescence kinetics to probe action target of 3-acetyl-5-isopropyltetramic acid. Environ Exp Bot..

