# Supplementary material for: Mechano-stimulated modifications in the chloroplast antioxidant system and proteome changes are associated with cold response in wheat
Source: BMC Plant Biol. 2015 Sep 11;15:219. doi: 10.1186/s12870-015-0610-6 (PMC4566287; doi:10.1186/s12870-015-0610-6)
Supplement: Additional file 1: Figure S1. — Temperature difference between low temperature treatment and the normal temperature control during the cold stress treatment at jointing. (DOCX 1175 kb) [file 12870_2015_610_MOESM1_ESM.docx]

**Supplementary information**

**Title:** Mechano-stimulated modifications in chloroplast antioxidant system and proteome changes are associated with cold tolerance in wheat

**Running title:** Mechano-stimulated cold tolerance in wheat

**Authors:** Xiangnan Li^1, 2^, Chenglong Hao^1^, Jianwen Zhong^1^, Fulai Liu^2^, Jian Cai^1^, Xiao Wang^1^, Qin Zhou^1^, Tingbo Dai^1^, Weixing Cao^1^, Dong Jiang^1^*

**Affiliations:** ^1^National Engineering and Technology Center for Information Agriculture / Key Laboratory of Crop Physiology and Ecology in Southern China, Ministry of Agriculture, Nanjing Agricultural University, Nanjing 210095, China

^2^University of Copenhagen, Faculty of Science, Department of Plant and Environmental Sciences, Højbakkegaard Allé 13, DK-2630 Taastrup, Denmark

Temperature (°C)

∆ Temperature (°C)

0 18 36 54 72 90 96

Hours of low temperature treatment (h)

Fig. S1 Temperature difference between low temperature treatment and the normal temperature control during the cold stress treatment at jointing.
